# Artificial Intelligence Chatbots and Their Responses to Most Searched Spanish Cancer Questions

**DOI:** 10.1002/cam4.71364

**Published:** 2025-11-10

**Authors:** En Cheng, Jesus D. Anampa, Carolina Bernabe‐Ramirez, Juan Lin, Xiaonan Xue, Carmen R. Isasi, Alyson B. Moadel‐Robblee, Edward Chu

**Affiliations:** ^1^ Department of Epidemiology and Population Health Albert Einstein College of Medicine Bronx New York USA; ^2^ Cancer Epidemiology, Prevention, and Control Program Montefiore Einstein Comprehensive Cancer Center Bronx New York USA; ^3^ Cancer Therapeutics Program Montefiore Einstein Comprehensive Cancer Center Bronx New York USA; ^4^ Department of Oncology Albert Einstein College of Medicine Bronx New York USA; ^5^ Department of Pediatrics Albert Einstein College of Medicine Bronx New York USA

**Keywords:** artificial intelligence, health literacy, Hispanic Americans, natural language processing, neoplasms, patient education

## Abstract

**Background:**

Artificial intelligence (AI) chatbots perform well in answering English cancer questions. For Spanish, their performance is unknown and may differ by free vs. paywall versions.

**Methods:**

We evaluated the quality (range: 1–5 points), actionability (range: 0–100%), and readability (range: 1–13 grades) of six popular AI chatbots in responding to the 15 most searched Spanish questions regarding breast, prostate, and colon cancer.

**Results:**

The quality of overall AI chatbot responses was good (mean [95% CI]: 3.5 [3.4–3.6] points), while the actionability was low (mean [95% CI]: 35.6% [30.8%–40.3%]). The readability was high‐school‐level (mean [95% CI]: 9.2 [8.8–9.6] grades), not concordant with the American Medical Association recommendation (≤ 6th grade). The quality, actionability, and readability did not differ by free and paywall versions (*p* > 0.05).

**Conclusion:**

Our findings suggested AI chatbots may generate good‐quality responses to Spanish cancer questions, regardless of free or paywall versions. However, further improvement in actionability and readability is needed to benefit Spanish‐speaking patients.

## Graphical abstract

Artificial intelligence (AI) chatbots are trained using English materials, and their performance in answering Spanish cancer questions is unknown. After comparing the six most popular AI chatbots, we found their answers were generally accurate (but hard to read) and needed more improvement in supporting patients to make cancer‐related decisions.

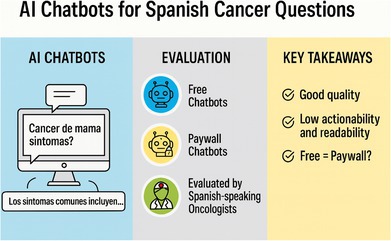

## 1 Introduction

After cancer diagnosis, 60% of patients in the US seek online information about cancer symptoms, diagnoses, and treatments [1], and artificial intelligence (AI) chatbots are increasingly recognized as a paradigm shift for patients to access such information [2]. Prior studies suggested that AI chatbots performed well in answering English cancer‐related questions [3, 4, 5, 6, 7], likely because they were predominantly trained on English materials [8, 9]. In addition to free versions, these AI companies also offer subscription‐based paywall versions claiming to provide enhanced capabilities. Thus, there have been increasing concerns that the linguistic imperialism of English and paywall barriers of AI chatbots may exacerbate existing disparities in cancer‐related health literacy for minority populations.

Spanish is the most spoken language after English in the US and spoken by 94% of US Hispanics/Latinos (the largest US minority population) [9]. Few studies have evaluated the performance of AI chatbot responses to Spanish cancer questions. Therefore, we utilized 6 popular AI chatbots (free and paywall versions) and assessed the quality, actionability, and readability of their responses to most searched Spanish cancer questions in the US.

## 2 Methods

This cross‐sectional study focused on the top 3 most common cancers diagnosed in US Hispanics/Latinos including *cancer de mama* (breast cancer), *cancer de prostata* (prostate cancer), and *cancer de colon* (*colon cancer*) [10]. From Google Trends (searching time range: from January 1, 2020 to January 1, 2024), we identified the top 5 most searched Spanish cancer questions in the US associated with these cancers (Supporting Information Methods and Table S1). On March 20, 2024, we put 15 searching‐related questions into free and paywall versions of ChatGPT (OpenAI Inc.), Claude (Anthropic PBC), and Gemini (Google LLC) available then: ChatGPT 3.5 (free), ChatGPT 4.0 (paywall), Claude 3 Sonnet (free), Claude 3 Opus (paywall), Gemini 1.0 (free), and Gemini 1.0 Advanced (paywall). ChatGPT, Claude, and Gemini were among the most commonly used AI chatbots when our study was conducted [11]. Out of 90 generated Spanish responses, we provided a sample for illustration (Table 1). This study obtained Institutional Review Board approval and followed the Standards for Reporting Qualitative Research (SRQR) reporting guideline.

**TABLE 1:** Examples of free and paywall versions of AI chatbot responses to a most searched spanish question regarding breast cancer (*cancer de mama*).

AI chatbot type	Response to “*Cancer de mama sintomas* (breast cancer symptoms)”
ChatGPT 3.5 (free)	Los síntomas del cáncer de mama pueden variar de una persona a otra, y algunas personas pueden no experimentar síntomas en absoluto. Sin embargo, algunos síntomas comunes del cáncer de mama incluyen: Presencia de un bulto o masa: Puede sentirse como un nódulo duro o una protuberancia en la mama o la axila. Cambios en el tamaño o la forma del seno: Esto puede ser evidente al comparar un seno con el otro. Cambios en la piel de la mama: Pueden incluir enrojecimiento, hinchazón, hoyuelos, arrugas o aspecto de cáscara de naranja. Cambios en el pezón: Esto podría manifestarse como cambios en la forma del pezón, retracción del pezón hacia adentro, enrojecimiento, descamación o secreción que no sea leche materna. Dolor en la mama: Aunque no todos los cánceres de mama causan dolor, algunas personas pueden experimentar molestias o sensibilidad en la mama. Cambios en la apariencia o sensación de la mama: Puede notar que una parte de la mama se siente diferente del resto, como más densa o firme. Es importante recordar que estos síntomas no siempre indican cáncer de mama, y muchas veces pueden ser causados por afecciones benignas. Sin embargo, cualquier cambio o síntoma inusual en la mama debe ser evaluado por un médico para determinar su causa y recibir un diagnóstico adecuado. La detección temprana puede mejorar significativamente las posibilidades de un tratamiento exitoso.
ChatGPT 4.0 (paywall)	Los síntomas del cáncer de mama pueden variar de una persona a otra, y en algunos casos, el cáncer de mama puede ser asintomático, especialmente en sus etapas iniciales. Sin embargo, hay varios síntomas y signos que pueden indicar la presencia de cáncer de mama. Estos incluyen: ***Bulto o engrosamiento en la mama o bajo el brazo*** **:** Un signo común de cáncer de mama es la aparición de un bulto o engrosamiento en la mama o debajo del brazo. Este bulto suele ser diferente del tejido mamario circundante y puede ser detectado mediante autoexamen o por un profesional de la salud durante un examen físico. ***Cambios en el tamaño, forma o contorno de la mama*** **:** Cualquier cambio notable en el tamaño, la forma o el contorno de una mama puede ser un indicador de cáncer de mama. ***Cambios en la piel de la mama*** **:** La piel de la mama puede mostrar cambios como enrojecimiento, hundimientos, o tener un aspecto similar a la piel de naranja (textura similar a la piel de esta fruta debido a la inflamación y bloqueo de los canales linfáticos). ***Secreción del pezón*** **:** La aparición de una secreción del pezón, especialmente si es sanguinolenta o de color inusual, puede ser un signo de cáncer de mama. No todas las secreciones son indicativas de cáncer, pero es importante que sean evaluadas por un médico. ***Cambios en el pezón*** **:** Los cambios en el pezón, como un pezón invertido (que se retrae hacia dentro) o cambios en la textura o el color del pezón o la areola, pueden ser síntomas de cáncer de mama. ***Dolor en la mama o el pezón*** **:** Aunque el cáncer de mama no siempre es doloroso, cualquier dolor persistente en la mama o el pezón debe ser evaluado. ***Irritación o bultos en la piel de la mama*** **:** La aparición de irritaciones, bultos, o áreas escamosas en la piel de la mama o el pezón pueden ser indicativos de ciertos tipos de cáncer de mama. Es importante recordar que estos síntomas no significan definitivamente que una persona tenga cáncer de mama, ya que muchos de ellos pueden ser causados por condiciones benignas. Sin embargo, si se detecta alguno de estos síntomas, es crucial consultar a un médico para realizar un diagnóstico y, si es necesario, comenzar el tratamiento lo antes posible. La detección temprana es clave para mejorar las posibilidades de tratamiento exitoso.
Claude 3 Sonnet (free)	Los síntomas más comunes del cáncer de mama incluyen: Bulto o protuberancia en el seno o la axila: ○ Suele ser el síntoma más frecuente ○ Puede ser duro, indoloro y con bordes irregulares Cambios en el tamaño o forma del seno: ○ Hinchazón parcial o completa del seno ○ Cambios en la textura de la piel (piel de naranja) Cambios en el pezón: ○ Pezón hundido o invertido ○ Secreción del pezón (puede ser acuosa, lechosa o sanguinolenta) ○ Enrojecimiento, descamación o formación de costras en el pezón Cambios en la piel del seno: ○ Enrojecimiento, descamación o formación de hoyuelos ○ Engrosamiento o hinchazón de una parte de la piel del seno Dolor en el seno o pezón: ○ Aunque el dolor no es un síntoma común, algunos tipos de cáncer de mama pueden causar molestias Es importante tener en cuenta que estos síntomas también pueden presentarse en condiciones benignas de la mama. Sin embargo, si notas algún cambio inusual, es recomendable consultar a un profesional médico para un examen y evaluación adecuados. La detección temprana a través del autoexamen mamario y las mamografías periódicas aumenta significativamente las probabilidades de un tratamiento exitoso.
Claude 3 Opus (paywall)	Los síntomas del cáncer de mama pueden variar de una persona a otra, y algunos tipos de cáncer de mama pueden no presentar síntomas evidentes en las primeras etapas. Sin embargo, los siguientes son algunos de los síntomas más comunes: Bulto o engrosamiento en el seno o la axila: Este es el síntoma más común. El bulto suele ser indoloro, duro y con bordes irregulares, aunque también puede ser blando y redondeado. Cambios en el tamaño o la forma del seno: Puede haber hinchazón de parte o de todo el seno, o bien una sensación de pesadez. Cambios en la piel del seno: La piel puede presentar hoyuelos, arrugas, enrojecimiento o aspecto de piel de naranja. Cambios en el pezón: El pezón puede retraerse o invertirse, o la piel del pezón puede volverse escamosa. Secreción del pezón: Puede haber una secreción que no sea leche materna, especialmente si es de un solo pezón y es sanguinolenta. Dolor: Aunque los bultos cancerosos suelen ser indoloros, pueden causar dolor en algunas ocasiones. Es importante recordar que estos síntomas también pueden ser causados por condiciones benignas. Sin embargo, si notas alguno de estos cambios, es crucial que consultes a un médico para un diagnóstico adecuado. La detección temprana es clave para un tratamiento exitoso del cáncer de mama.
Gemini 1.0 (free)	Es importante estar familiarizada con los posibles síntomas del cáncer de mama para que pueda detectarlo lo antes posible. La detección temprana del cáncer de mama es fundamental para un tratamiento eficaz. Aquí están algunos de los síntomas más comunes del cáncer de mama: Un bulto nuevo en la mama o la axila (debajo del brazo). 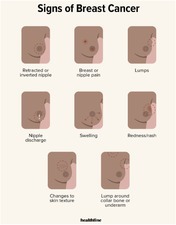 (https://www.healthline.com/health/breast‐cancer/warning‐signs#early‐signs) Engrosamiento o hinchazón de una parte de la mama. Irritación o piel con hoyuelos en la mama. Enrojecimiento o descamación en la zona del pezón o la mama. Hundimiento del pezón o dolor en esa zona. Secreción del pezón, que no sea leche, incluso de sangre. Cualquier cambio en el tamaño o la forma de la mama. Dolor en cualquier parte de la mama. Es importante tener en cuenta que estos síntomas también pueden ser causados por otras afecciones benignas (no cancerosas). Pero si experimenta alguno de estos síntomas, es importante que consulte a su médico para que lo revise. La Sociedad Americana del Cáncer [American Cancer Society] recomienda que todas las mujeres con un riesgo promedio de cáncer de mama se realicen una mamografía de detección a partir de los 40 años. Una mamografía es una radiografía de la mama que puede detectar cambios en el tejido mamario que podrían ser cancerosos. Sources asociacionale.org.mx/10‐datos‐que‐debes‐conocer‐sobre‐el‐dia‐mundial‐de‐la‐lucha‐contra‐el‐cancer‐de‐mama/ www.tunuyan.gov.ar/site/el‐municipio‐concientizo‐sobre‐la‐prevencion‐de‐cancer‐de‐mam/
Gemini 1.0 advanced (paywall)	Aquí te detallo los síntomas más comunes del cáncer de mama. Es importante recalcar que estos pueden variar entre personas, y que algunas podrían no presentarlos en las etapas iniciales de la enfermedad. ***Síntomas frecuentes del cáncer de mama*** **:** Bulto o engrosamiento: Un bulto nuevo en la mama o axila que se siente diferente al tejido de alrededor. Puede ser indoloro o sensible. Cambio de tamaño o forma de la mama: Aumento de tamaño o hinchazón de una parte de la mama, incluso si no se detecta un bulto específico. Cambios en la piel de la mama: Enrojecimiento, descamación, hoyuelos (con aspecto similar a la piel de naranja), irritación, o arrugas en la piel de la mama. Cambios en el pezón: Retracción (el pezón se hunde), dolor, enrojecimiento, descamación o secreción inusual (que no sea leche materna), especialmente si es sanguinolenta. Dolor: Si bien no es el síntoma más común, puedes sentir dolor en la mama o el pezón de forma inusual. ***¿Qué hacer si noto algún síntoma?*** Si detectas alguno de estos síntomas, es crucial que consultes con tu médico lo antes posible. Aunque no siempre indican cáncer de mama, es importante hacer una evaluación profesional para descartar cualquier problema grave. La detección temprana es vital para mejorar las probabilidades de un tratamiento exitoso. ***¡Recuerda!*** La autoexploración mamaria es importante para familiarizarte con tus senos y notar cambios. Sin embargo, no sustituye las mamografías regulares, que son la mejor forma de detectar el cáncer de mama en estadios tempranos cuando aún es muy tratable.

For each response, the quality was assessed using the DISCERN Instrument (16 questions; an overall score ranging from 1 [low quality] to 5 [high quality]) [12], actionability using the Patient Education Materials Assessment Tool (7 questions; an overall score ranging from 0 [no clear action suggestions] to 100% [clear action suggestions]) [13], readability using the Fernández Huerta Reading Grade Level (calculated using a mathematical formula; an overall score ranging from 1 [1st grade] to 13 [college]) [14]. Particularly, actionability represented the degree to which AI chatbot responses may help individuals with diverse backgrounds and varying levels of health literacy in identifying specific actions to take.

Two board‐certified oncologists (J.D.A. and C.B.‐R.) speaking native Spanish were blinded to AI chatbots and independently assessed responses. Raters completed a robust rater training process including (1) an overview of research purpose and specific aims; (2) completing a didactive review of assessment tools; (3) sample practice and calibration; (4) inter‐rater reliability assessment; (5) discussing common challenges and conflict resolution; and (6) following standard operating procedures to assess all responses. In the phase of pilot testing, we used the most searched Spanish questions for liver cancer (*cancer de higado*) as an example and generated the related responses for raters' practice. Any discrepancies were referred to another investigator (E.C.) and discussed among these three investigators to reach consensus. Each oncologist involved in this study for rating responses had more than 10 years of experience in clinical practice, and the intraclass correlation coefficient (ICC) was 0.91 suggesting excellent agreement [15].

To compare free vs. paywall versions across AI chatbots, we utilized linear mixed‐effect models to assess their differences of quality, actionability, and readability (Supporting Information Methods). To compare free vs. paywall versions within each type (ChatGPT, Claude, or Gemini), we used the paired t‐test instead. Estimates were presented as means with 95% confidence intervals (CI), and two‐sided *p* < 0.05 was considered statistically significant. All analyses were conducted using R (Version 4.3.1) from September 27, 2024, to October 16, 2024.

## 3 Results

**FIGURE 1:**
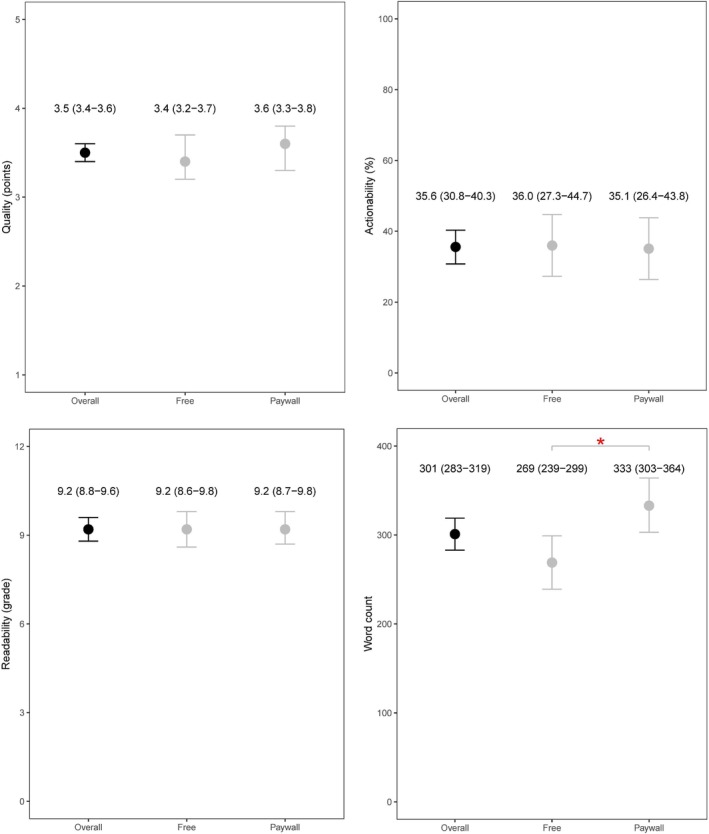
The comparisons (means [95% confidence intervals]) of free versus paywall versions in responding to most searched spanish cancer questions. Dots refer to means, and the error bars refer to 95% confidence intervals. The values (means [95% confidence intervals]) for overall, free, and paywall versions were provided in the plots. The symbol * indicates the difference as statistically significant (*p* < 0.001).

Of 90 AI chatbot responses (Figure 1), the quality of overall AI chatbot responses was good (mean [95% CI]: 3.5 [3.4–3.6] points), although the actionability was low (mean [95% CI]: 35.6% [30.8%–40.3%]). The readability was at the high school level (mean [95% CI]: 9.2 [8.8–9.6] grade), and the average length of responses was 301 (95% CI: 283–319) words. The performance of quality, actionability, and readability did not differ significantly by free and paywall versions (*p* > 0.05), although paywall versions provided lengthier responses (*p* < 0.001).

Similar findings were observed within each AI type (Table S2). However, the paywall version of ChatGPT performed worse than its free version in the actionability (mean [95% CI]: 22.7% [10.2%–35.1%] vs. 44.0% [33.6%–54.4%]; *p* = 0.006). In contrast, a moderate increase in actionability was observed for the paywall version of Claude (*p* = 0.02), although its actionability remained low (mean [95% CI]: 33.3% [21.7%–44.9%]). Despite such differences, six AI chatbots provided generally accurate information for most searched Spanish cancer‐related questions. The responses were not readily actionable and were written at the high‐school level, which was not concordant with the American Medical Association (AMA) recommendation (6th grade or lower). Their performance did not improve markedly by using paywall versions.

## 4 Discussion

We were initially concerned about the performance of AI chatbot responses to Spanish cancer questions: AI chatbots were predominantly trained on English materials, due to the abundance and accessibility of English corpora in biomedical science as well as technology, literature, and everyday communications [8, 9]. However, AI chatbots (ChatGPT 3.5 and 4) were reported to pass the Spanish Medical Residency Entrance Examination (MIR) and achieve 100% correctness in the Oncology Specialty section, although only four oncology questions were tested in the MIR [16]. Our study found that six popular AI chatbots generated good‐quality Spanish responses (mean [95% CI]: 3.5 [3.4–3.6] points), and such a score was comparable to prior studies of AI chatbots in responding to English cancer questions [3, 4, 5, 6, 7].

Despite good quality, we observed low actionability that may affect decision‐making and clinical actions among Hispanics/Latinos, such as seeking healthcare for cancer‐related symptoms and adhering to cancer treatment. Such low actionability was also observed in responding to English cancer question [3], suggesting that AI chatbots may need general improvement in helping patients take appropriate actions for cancer management. Interestingly, we noticed the ChatGPT paywall version performed worse than its free version in actionability. Although it may be a chance finding, there is another possible explanation: a paywall version typically runs a larger and superior AI model requiring stronger medical‐safety guardrails, which may consequently result in lower actionability [17]. In addition, we found the responses were written at high‐school‐level readability and exceeded the AMA recommendation of sixth grade or lower, especially considering that 7 in 10 foreign‐born Hispanics/Latinos finished high school or less [18]. This gap is concerning because patient care‐seeking behaviors and adherence to clinical recommendations can be notably affected by patient‐facing materials [19]. Further improvement in readability can move towards reducing cancer health disparities and health literacy barriers among Hispanics/Latinos.

Initially, we were also concerned about the paywall as another barrier to accessing the most advanced AI technologies, especially for low‐income Hispanics/Latinos. However, we did not find that paywall versions markedly outperform free versions, although they generated lengthier responses. Our findings aligned with a prior study of using free and paywall ChatGPT versions in answering English cancer questions [5], and text simplification should be considered in AI technology development meanwhile ensuring accurate and actionable information to answer cancer questions.

In addition to answering cancer questions, there is increasing interest in applying AI chatbots to a variety of healthcare‐related domains (such as academic writing in biomedical research, mental health assessments, health promotion, and nutrition). However, the performance of AI chatbots is not always satisfactory and may vary across these domains, highlighting the need for supervision and improvement from both healthcare professionals and AI developers.

To our knowledge, this is the first study to evaluate the performance of AI chatbots in responding to the most searched Spanish cancer questions. One unique strength of our study is that board‐certified oncologists speaking native Spanish independently assessed all responses. However, our study has several limitations. First, we limited our study to the most searched Spanish questions in the US according to Google Trends, because such most searched queries were not publicly available by AI chatbots. Second, we primarily focused on the top 3 most common cancers in US Hispanics/Latinos, and future studies could consider a full spectrum of cancer types. Third, AI chatbots are rapidly evolving and their performance may be improved via using appropriate prompts [5], and thus future studies should consider testing more AI chatbots and a variety of prompts that may help Spanish‐speaking patients get better responses.

## 5 Conclusion

In conclusion, our findings suggested AI chatbots have the potential to generate good‐quality responses to Spanish cancer questions, regardless of free or paywall versions. However, their responses need improvement in actionability and readability to further benefit Spanish‐speaking patients with cancer. In the era of digital health, further research is needed to investigate the impact of AI chatbot implementation on health disparities across different minority populations.

## Author Contributions

En Cheng: conceptualization, data curation, funding acquisition, formal analysis, investigation, methodology, project administration, resources, software, supervision, validation, visualization, writing – original draft, writing – review and editing. Jesus D. Anampa: data curation, methodology, project administration, resources, writing – review and editing. Carolina Bernabe‐Ramirez: data curation, methodology, project administration, resources, writing – review and editing. Juan Lin: data curation, formal analysis, software, visualization, writing – review and editing. Xiaonan Xue: data curation, funding acquisition, formal analysis, software, visualization, writing – review and editing. Carmen R. Isasi: funding acquisition, writing – review and editing. Alyson B. Moadel‐Robblee: writing – review and editing. Edward Chu: funding acquisition, supervision, writing – review and editing.

## Ethics Statement

This study obtained Institutional Review Board's approval from Albert Einstein College of Medicine (Ethics Approval Number: 2024‐15752).

## Consent

The authors have nothing to report.

## Conflicts of Interest

The authors declare no conflicts of interest.

## Supporting information


**Supporting Information Methods.** Linear mixed‐effect models.
**Table S1:** Top 5 most searched Spanish cancer questions in the US extracted from Google Trends (from January 1, 2020 to January 1, 2024).
**Table S2:** The comparison of free vs. paywall versions within each AI chatbot type.

## Data Availability

De‐identified data may be requested from En Cheng (en.cheng@einsteinmed.edu) and Juan Lin (juan.lin@einsteinmed.edu). A review process includes verifying the availability of data, conducting a review of any existing agreements that may have implications for the project, and ensuring that any transfer is in compliance with the Institutional Review Board. The investigator will be required to sign a data release form prior to the transfer.
