# Supplementary material for: Artificial Intelligence Chatbots and Their Responses to Most Searched Spanish Cancer Questions
Source: Cancer Med. 2025 Nov 10;14(21):e71364. doi: 10.1002/cam4.71364 (PMC12599334; doi:10.1002/cam4.71364)
Supplement: Supplementary file 1 — Supporting Information Methods. Linear mixed‐effect models. Table S1: Top 5 most searched Spanish cancer questions in the US extracted from Google Trends (from January 1, 2020 to January 1, 2024). Table S2: The comparison of free vs. paywall versions within each AI chatbot type. [file CAM4-14-e71364-s001.docx]

**Supplementary Methods.** Top 5 Most Searched Spanish Cancer Questions from Google Trends

On the website of Google Trends (https://trends.google.com/trends/), the section “Explore” can provide most searched queries according to the location, timeframe, and terms of interest. In this study, we selected “United States” as the location, chose “1/1/2020-1/1/2024” the timeframe, and entered each cancer type (such as *cancer de mama*) as a term of interest, which generated a list of most searched questions regarding this cancer type. We then identified top 5 most searched Spanish cancer questions in the US associated with each cancer type.

**Supplementary Methods.** Linear Mixed-Effect Models

Linear Mixed-Effect Models to Assess the Performance of AI Chatbots to Most Spanish Cancer Questions

Y_ijk_ = α_i_ + β_1_X_j(i,k)_ + β_2_Z_k(i,j)_ + β_3_X_j(i,k)_ × Z_k(i,j)_ + ε_ijk_

Y_ijk_: Outcome of the i-th question for the j-th version of the k-th AI type

X: AI subscription version (Free, Paywall)

Z: AI chatbot type (ChatGPT, Claude, Gemini)

α_i_ ~ N (0, σ^2^)

ε_ijk_ ~ N (0, σ_ε_^2^)

Reference

1. Harrell FE Jr: Regression Modeling Strategies: With Applications to Linear Models, Logistic and Ordinal Regression, and Survival Analysis. New York, NY, Springer, 2015.

2. Gałecki A, Burzykowski T: Linear Mixed-Effects Models Using R: A Step-by-Step Approach. New York, NY, Springer, 2013.

**Supplementary Table 1**. Top 5 Most Searched Spanish Cancer Questions in the US Extracted from Google Trends (From January 1, 2020 to January 1, 2024)

| Top | *Cancer de mama* (breast cancer) | *Cancer de prostata* (prostate cancer) | *Cancer de colon* (colon cancer) |
| --- | --- | --- | --- |
| 1 | *El cancer de mama* | *Sintomas cancer de prostata* | *Sintomas de cancer de colon* |
| 2 | *Cancer de mama sintomas* | *Sintomas de prostata* | *Sintomas de cancer* |
| 3 | *Sintomas de cancer* | *Cancer en la prostata* | *Cancer del colon* |
| 4 | *Fotos de cancer de mama* | *Que es la prostata* | *Cancer de estomago* |
| 5 | *Cancer de mama en hombres* | *Prostata inflamada* | *Que es el cancer* |

**Supplementary Table 2**. The Comparison of Free vs. Paywall Versions Within Each AI Chatbot Type

| Performance^a^ | ChatGPT | | | Claude | | | Gemini | | |
| --- | --- | --- | --- | --- | --- | --- | --- | --- | --- |
|  | Free | Paywall | *P* | Free | Paywall | *P* | Free | Paywall | *P* |
| Quality (points) | 3.3 (2.9-3.8) | 3.6 (3.3-3.9) | 0.10 | 3.5 (3.2-3.8) | 3.5 (3.2-3.8) | 1.00 | 3.5 (3.2-3.8) | 3.6 (3.3-3.9) | 0.33 |
| Actionability (%) | 44.0 (33.6-54.4) | 22.7 (10.2-35.1) | 0.006 | 20.0 (8.9-31.1) | 33.3 (21.7-44.9) | 0.02 | 44.0 (30.6-57.4) | 49.3 (41.1-57.6) | 0.41 |
| Readability (grade) | 10.9 (10.0-11.8) | 9.7 (8.8-10.6) | 0.06 | 8.9 (8.0-9.8) | 9.1 (8.1-10.2) | 0.62 | 7.9 (7.2-8.6) | 8.8 (8.1-9.6) | 0.10 |
| Word count | 288 (245-330) | 383 (336-429) | <0.001 | 148 (90-205) | 299 (256-341) | <0.001 | 300 (247-353) | 319 (282-357) | 0.47 |

^a^ Data are presented as means (95% confidence intervals)
